# Protective effect of Quercetin in the Regression of Ethanol-Induced Hepatotoxicity

**DOI:** 10.4103/0250-474X.58186

**Published:** 2009

**Authors:** A. Vidhya, M. Indira

**Affiliations:** Department of Biochemistry, University of Kerala, Kariavattom, Thiruvananthapuram-695 581, India

**Keywords:** Quercetin, ethanol, hepatotoxicity, serum amino transferases, lipid peroxidation

## Abstract

This study examined the protective effects of quercetin on chronic ethanol-induced liver injury. Rats were treated with ethanol at a dose of 4 g/100 g/day for 90 days. After ethanol intoxication, levels of serum amino transferases were significantly elevated. Decreased activity of superoxide dismutase, catalase, glutathione peroxidase and glutathione reductase was also observed on ethanol administration. Increased amounts of lipid peroxidation products viz. hydroperoxides, conjugated dienes and malodialdehyde were observed on ethanol intoxication. Ethanol administration resulted in significant decrease in liver glutathione content. After 90 days, the control animals were divided into two groups, the control group and the control+quercetin group. Ethanol-treated group was divided into two groups, abstention group and quercetin-supplemented group. After 30 days, the animals were sacrificed and various biochemical parameters were analyzed. The changes in enzyme activities as well as levels of lipid peroxidation products were reversed to a certain extent by quercetin. Quercetin supplementation resulted in increase of glutathione content to a significant level compared to normal abstention group. Quercetin supplemented group showed a faster recovery than abstention group. This shows the protective effect of quercetin against chronic ethanol induced hepatotoxicity. Histopathological study is also in line with these results.

Alcohol abuse and its medical and social consequences are a major health problem in many areas of the world. Ethanol exerts its effect either directly or through derrangements in metabolic, hormonal and nutritional mechanisms[1]. Excessive generation of free radicals play an important role in alcohol induced cellular damage[2]. Ethanol abuse affects many organ systems most notably the liver causing both acute and chronic liver disease and the central nervous system[3]. A large number of studies are in progress aiming to identify substances that would be effective in reducing the severity of alcoholic liver disease. Although removal of alcohol still represents the most effective intervention to prevent the manifestation of alcoholic liver disease, trials are underway aiming a faster recovery.

It was established that generation of reactive oxygen species is enhanced after ethanol intoxication, and that they play a major role in the creation of oxidative stress, which may additionally be enhanced by the depletion in the antioxidant defense system and, in consequence, by an imbalance between prooxidants and antioxidants[4]. Aim of our study was to induce hepatotoxicity using ethanol and then to assess the reversal of damage once ethanol consumption is stopped.

Flavanoids are a group of naturally occuring compounds widely distributed as secondary metabolites in plant kingdom. One of these flavanoids, quercetin (3,5,7,3,4-pentahydroxy flavon), is one of the most prominent dietary antioxidant. The ability of quercetin to scavenge highly reactive species such as peroxynitrite and the hydroxy radical is suggested to be involved in these possible beneficial health effects[5]. Tieppo *et al*.[6] has stated that quercetin increased the genomic stability in the cirrhotic rats, suggesting beneficial effects, probably by its antioxidant properties[7]. Quercetin is also known to act as an effective antioxidant by chelating metal ions and/or scavenging free radicals[8]. Several studies have demonstrated that quercetin enhanced the antioxidative defense system by upregulating antioxidant enzymes[9].

One of the most important problems faced by those who have stopped alcohol consumption after chronic intake, is the recovery of damaged hepatocytes to normal level. Liver regeneration occurs normally in damaged liver. But the time required varies depending upon the severity of damage, which inturn is determined by the dose and duration of alcohol intake.

There are hardly any studies reported in the literature regarding the protective effect of quercetin in ethanol-induced toxicity. It has been proved that oxidative stress is the mechanism behind ethanol-induced toxicity and quercetin is shown to have beneficial effect on hepatic oxidative stress. Hence, we have studied the effect of quercetin in the regression of ethanol-induced hepatotoxicity.

## MATERIALS AND METHODS

Male Sprague Dawley rats weighing between 150 and 200 g were used. Animals were housed in polypropylene cages. Cages were kept in a room that was maintained between 28 and 32° and a 12 h dark and light cycle was maintained. The study protocol was approved by the Institutional Animal Ethics Committee. Animals were handled using the laboratory animal welfare guidelines. Rats were fed with rat feed (Lipton India Ltd). Water was given *ad libitum*. Quercetin was purchased from M/s SRL Ltd., Mumbai, India and ethanol from M/s Merck Ltd, Mumbai, India

**Fig. 1:**
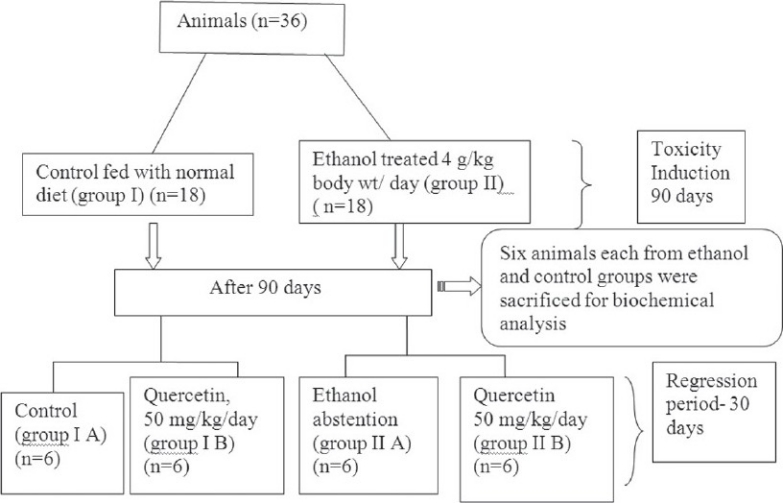
Experimental design n=number of animals

A total of 36 animals were divided into two groups, group I was the control group fed with normal diet and group II was the ethanol group (4 g ethanol/kg/day). Ethanol diluted with distilled water (1:1) was given orally by gastric tube. After 90 days, ethanol administration was stopped and 6 animals each from control group and ethanol group were sacrificed after overnight fasting and liver was collected for biochemical analysis. The rest of animals in control group were divided into two groups, group I A, control group fed with normal diet and group I B, quercetin-supplemented group (50 mg quercetin/kg/day). Quercetin was freshly dissolved in distilled water during treatment and given orally by gastric tube. The animals in the ethanol group were also divided into two groups, group II A, abstention group and group II B, quercetin-supplemented group (50 mg quercetin/kg/day) for 30 days. Group I was given isocaloric glucose solution. This is schematically represented in the fig. 1. At the end of the experimental period, animals were sacrificed after overnight fasting. The liver was dissected out and cleaned with ice-cold saline, blotted dry and immediately transferred to ice-cold container for various evaluations.

### Biochemical methods:

Activities of super oxide dismutase (SOD, EC.1.15.1.1) and catalase (EC.1.11.1.6) were determined by the method of Kakkar *et al*.[10] and Maehly and Chance[11], respectively. The activity of glutathione peroxidase (GPx, EC.1.11.1.9) was determined by Lawerence and Burk as modified by Agergurd and Jence[12]. The activity of glutathione reductase (GR) was determined by the procedure of David and Richard[13]. Protein was estimated by the method of Lowry *et al*.[14]. The activities of aspartate amino transferase (AST) and alanine amino transferase (ALT) were determined by the method of Reitman and Frankel as described by Wooten[15]. Malondialdehyde (MDA) was estimated by the method of Ohkava[16]. Hydroperoxides were estimated by the method of Mair and Hall[17] and conjugated dienes (CD) were estimated by the method of Recknagel and Ghoshal[18]. Glutathione content in tissue was estimated by the method of Beutler *et al*[19].

### Statistical analysis:

The results were analysed using a statistical programme SPSS/PC+, version 10 (SPSS Inc. Chicago, IL, USA). A one way ANOVA was employed for comparison among the six groups. Duncan's post hoc multiple comparison test of significant difference among groups were determined. p≤ 0.05 was considered significant.

### Histopathological studies:

For histopathological studies, liver was fixed in Bauins' fixative and sections were taken in the microtome. Sections were stained using haematoxylin and eosin. Pathological changes were examined using a sensitive light microscope.

## RESULTS AND DISCUSSION

Activities of AST, ALT and GGT in serum and liver significantly increased in rats given ethanol (Table 1). Activities of these enzymes were decreased in group II A in comparison with group II. Supplementation of quercetin in group II B further reduced the activities of these enzymes. The activities of antioxidant enzymes SOD and catalase were found to be significantly decreased in tissues of rats exposed to ethanol (Table 2). The activities of these enzymes were considerably increased in quercetin supplemented group in comparison with those fed ethanol alone.

**TABLE 1:** TOXICITY MARKER ENZYMES

Group	ALT (μmoles of pyruvate liberated /min/mg protein)		AST (μmoles of OAA liberated/min/mg protein)	
		
	Liver	serum	Liver	serum
I	16.38±1.84	193.13±18.52	15.42±1.47	52.51± 4.6
II	96.85±8.41^a^	534.11±48.64^a^	61.12±5.57^a^	144.83±12.8^a^
I A	16.87±1.86	201.32±19.1	16.12±1.56	54.82±4.8
I B	21.44±1.96	191.35±17.39	16.29±1.55	48.42±3.1
II A	60.77±5.54^b^	499.67±48.62^b^	49.31±4.71^b^	112.09±9.2^b^
II B	49.22±4.86^c^	342.88±32.88^c^	34.48±3.26^c^	78.74±5.9^c^

Values expressed as mean±SD of six rats.

a -*P*<0.05 between control and ethanol treated groups

b - *P*<0.05 between ethanol treated and abstention groups

c - *P*<0.05 between abstention group and quercetin supplemented group.

**TABLE 2:** ACTIVITY OF ANTIOXIDANT ENZYMES IN LIVER

Group	SOD (#units/ mg protein)	Catalase (*units /mg protein)	GPx (μmole NADPH oxidised/min)	GR (μmole NADPH oxidised/min)
I	82.67±8.34	64.21±6.15	12.30±1.17	20.98±2.02
II	20.11 ±1.83^a^	37.57±3.60^a^	4.48±0.42^a^	3.80±0.41^a^
I A	84.56±8.36	62.41± 5.9	11.92±1.06	19.88±1.86
I B	74.42±6.76^b^	51.56±4.94^b^	9.53±0.86^b^	6.06±0.58^b^
II A	31.93±3.07^c^	45.60±4.61^c^	5.82±0.62^c^	8.83±0.80^c^
II B	42.11±3.83^d^	58.09±5.57^d^	7.21±0.65^d^	15.02±1.36^d^

Values expressed as mean±SD of six rats.

* units-velocity constant/sec.

# units- enzyme concentration required to inhibit the chromogen production by 50% in 1 minute.

a - *P*<0.05 between control and ethanol treated groups

b - *P*<0.05 between ethanol treated and abstention groups

c - *P*<0.05 between control and control+ quercetin group

d -*P*<0.05 between abstention group and quercetin supplemented group.

The activity of glutathione peroxidase was reduced in ethanol-treated group (Table 2). The activity of this enzyme was significantly enhanced in quercetin supplemented group. The activity of glutathione reductase was lowered in ethanol treated group (Table 2). The activity of this enzyme was significantly enhanced in the liver of quercetin supplemented group when compared to other groups.

Alcohol induced significant lipid peroxidation (Table 3). Level of malondialdehyde was increased significantly in ethanol treated group. This was significantly reduced by quercetin supplementation. Upon alcohol administration; there was significant increase in tissue hydroperoxide level. This was reduced significantly by quercetin supplementation. Conjugated diene level was also found to be higher in ethanol treated group. Quercetin supplementation caused a reduction in conjugated diene level compared to abstention group. Ethanol administration resulted in significant decrease in liver glutathione content. Quercetin supplementation resulted in increase of glutathione content to a significant level compared to normal abstention group (Table 4).

**TABLE 3:** CONCENTRATION OF LIPID PEROXIDATION PRODUCTS IN LIVER

Group	MDA (mM/100g wet tissue)	Hydroperoxides dienes (mM/100g wet tissue)	Conjugated (mM/ 100g wet tissue)
I	0.70±0.067	8.83±0.85	69.01±6.23
II	1.67±0.16^a^	23.64±2.09^a^	213.78±19.53^a^
I A	0.68±0.064	8.86±0.87	70.42±6.81
I B	0.63±0.06	7.80±0.68	66.31±6.36
II A	1.37±0.12^b^	18.10±1.32^b^	196.25±17.72^b^
II B	1.10±0.11^c^	12.82±1.17^c^	134.59±14.47^c^

Values expressed as mean±SD of six rats.

a -*P*<0.05 between control and ethanol treated groups

b - p<0.05 between ethanol treated and abstention groups

c - *P*<0.05 between abstention group and quercetin supplemented group.

**TABLE 4:** GLUTATHIONE CONTENT LEVEL IN LIVER

Group	Glutathione content (mg/100g wet tissue)
I	572.46±52.51
II	391.06± 35.68^a^
I A	576.36±52.68
I B	542.25±47.92
II A	412.01±37.99^b^
II B	482.16± 41.34^c^

Values expressed as mean±SD of six rats.

a - *P*<0.05 between control and ethanol treated  groups

b -  *P*<0.05  between  ethanol  treated  and  abstention  groups

c - *P*<0.05 between abstention group and quercetin supplemented group.

**Fig. 2:**
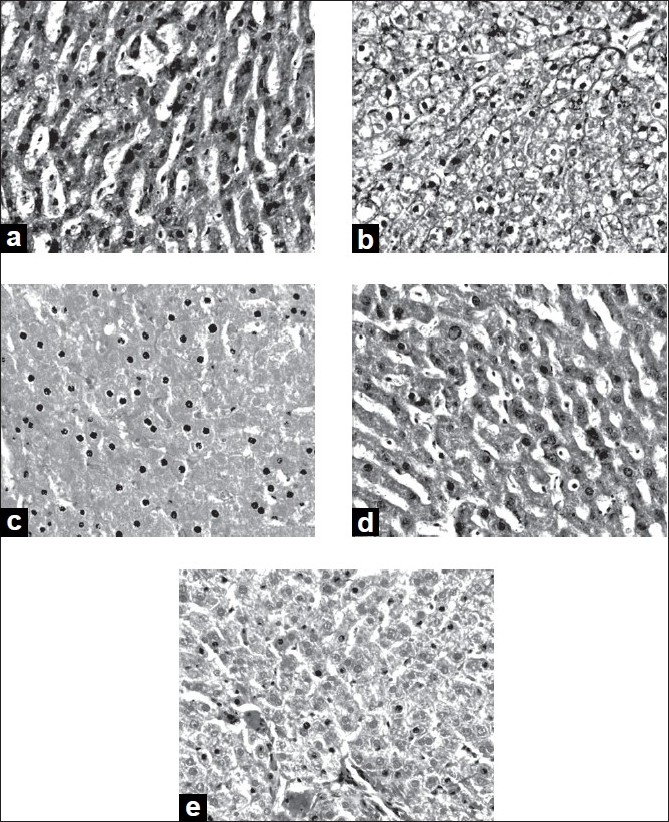
Histopathological sections of liver Microphotograph of the liver of the different treatment groups. Magnification 40 X, H and E staining. a) Ethanol-treated group- Damage is noted in the slide as necrosis, ballooning of hepatocytes, steatosis, vacuolization and dilation of sinusoids; b) Control group showing the structure of normal liver. Normal hepatocytes are seen. c) Control+quercetin-treated group, shows hepatocytes with normal structure. d) Ethanol-abstention group, marked damage to hepatocytes is noted. Sinusoidal dilation is seen. e) Ethanol+quercetin-treated group, the damage is of lesser degree compared to ethanol group. Mild ballooning of hepatocytes is noted.

After ethanol administration liver sections showed extensive hepatocellular damage as evidenced by ballooning of hepatocytes, steatosis, vacuolization and dilation of sinusoids (fig. 2a). The histological features of the liver in the control group showed a normal liver architecture and cell structure (fig. 2b). Quercetin supplementation to control rats did not alter normal liver structure (fig. 2c). Abstention from ethanol resulted in reduction in hepatic damage as evidenced by mild steatosis (fig. 2d). The histopathological changes were ameliorated by quercetin treatment. Damage is of lesser degree in quercetin-treated group (fig. 2e).

Ethanol treatment caused significant increase in AST and ALT activities, an indication of hepatocellular damage in rats. Treatment with quercetin reduced ethanol-induced toxicity as indicated by drop in activities of marker enzymes. In alcohol intoxication, as a result of structural changes, an increase in membrane permeability to ions has been demonstrated in different animal models[20]. The increase in membrane permeability causes translocation of ALT and AST into blood circulation as shown by abnormally high level of serum hepatic markers.

Antioxidant enzymes such as SOD, CAT and GP_X_ comprise a major supportive group of protection against free radicals. Chronic ethanol treatment caused significant decrease in SOD, CAT and GP_X_. There are reports to indicate that there is significant decrease in glutathione peroxidase in chronic ethanol abuse[21]. This is in agreement with our results. However, supplementation of quercetin increased GP_x_ activity significantly.

SOD prevents the inhibition of GP_X_ and CAT by scavenging superoxide radicals and GP_x_ and CAT in turn prevent the inhibition of SOD by scavenging H_2_O_2_[22]. In quercetin treated animals, SOD activity is increased, which could be due to the increase in glutathione peroxidase activity that lowered the levels of H_2_O_2_, thereby preventing the retro-inhibition on SOD. This is in accordance with earlier reports[23]. Quercetin supplementation attenuated all alterations in antioxidant enzymes- SOD, CAT, GP, GR, reduced glutathione in ethanol treated animals. Quercetin shows beneficial effects on liver damage by enhancing antioxidant enzyme activity and decreasing prooxidant effect[24]. This is due to the ability of quercetin to interact with hydroxyl, superoxide, alkoxyl and peroxyl radicals thereby subsequently scavenging them. Quercetin supplementation led to a slight decrease in antioxidant defense in normal controls. This may be due to prooxidant effect of quercetin in normal cells. Choi *et al*. had observed earlier that quercetin acts as a prooxidant in normal rats[25].

Glutathione constitutes the first line of defense against free radical and is a determinant of tissue susceptibility to oxidative damage. Quercetin significantly increased the reduced glutathione levels in alcohol intoxication. It has been reported that quercetin corrects the reduction in glutathione concentration and partially prevented the increase in collagen concentration, TBARS and GSSG/GSH ratio[26].

In the present study we observed that there was significant increase in lipid peroxidation products in ethanol consumption as shown by earlier studies[27]. The increased concentration of MDA, hydroperoxides and conjugated dienes, an index of lipid peroxidation may be due to increased production of free fatty acids which may serve as substrates for lipid peroxidation[28]. Quercetin also increased serum albumin and hepatic glutathione levels and reduced the hepatic level of malondialdehyde[29].

Previous studies have demonstrated that quercetin intake prevented and protected the liver from the oxidative damage induced by the ingestion of ethanol[30]. Effects and diminished liver injury by quercetin may be partially related to preservation of antioxidant defenses. The flavonoid has been shown to scavenge highly reactive species implicated in the peroxidation process such as oxygen and hydroxyl radicals.

Hepatotoxicity induced by ethanol is further confirmed by abnormal histological findings. Toxicity manifestations by ethanol in the liver tissue are revealed by morphological changes such as inflammation around portal triad (Triaditis) with severe fatty degeneration. Quercetin supplementation resulted in a faster recovery of normal cell structure compared to abstention group.

From these observations it can be concluded that quercetin offers protective effect against ethanol induced hepatotoxicity by attenuating lipid peroxidation, by scavenging free radicals and by enhancing the activity of antioxidants, which in turn detoxify free radicals. It can be developed as a drug for the reversal of chronic ethanol-induced hepatotoxicity.
